# Supplementary material for: Effects of growth rate, size, and light availability on tree survival across life stages: a demographic analysis accounting for missing values and small sample sizes
Source: BMC Ecol. 2015 Feb 28;15:6. doi: 10.1186/s12898-015-0038-8 (PMC4465470; doi:10.1186/s12898-015-0038-8)
Supplement: Additional file 1: — a. Goodness of fit testing: The output of the bootstrapping exercise on the fully parameterised model (ϕ t p c ) to determine model goodness of fit and to estimate the quasi-likelihood parameter used in subsequent analyses of survival. b. The output of the bootstrapping exercise on the simplified model to determine model goodness of fit and to estimate the quasi-likelihood parameter used in subsequent analyses of survival. c. Model output for each of the 17 models run for each tree species. The models are sorted by quasi-AICc (QAICc). For ACERCA and BETUSP no saplings were recorded and thus models 2, 3, 6, 9, 10, 11, 12, 13, 14, and 17 (as described in Table 1) could not be fitted. [file 12898_2015_38_MOESM1_ESM.doc]

**Supplement 1a.**

Goodness of fit testing: The output of the bootstrapping exercise on the fully parameterised model (φ t p c ) to determine model goodness of fit and to estimate the quasi-likelihood parameter used in subsequent analyses of survival.

|  |  |  | Goodness of fit results estimated from 1000 bootstrap simulations | | | |  |
| --- | --- | --- | --- | --- | --- | --- | --- |
|  |  | Observed model deviance | Mean model deviance | Median model deviance | Range of model deviance | Probability observed deviance does not differ from simulation | Estimated ^c |
| ACERCA* | φ c p c | 3.72 | 1.01 | 0.00 | 0 – 6.47 | 0.09 | 3.69 |
| ACERPS | φ t p c | 16.92 | 5.40 | 4.46 | 0 – 24.53 | 0.002 | 3.13 |
|  | φ c p c | 21.33 | 7.94 | 7.29 | 0 – 24.04 | 0.003 | 2.69 |
| BETUSP* | φ c p c | 6.34 | 4.42 | 4.20 | 0 – 13.35 | 0.090 | 1.43 |
| CORYAV | φ t p c | 59.73 | 18.50 | 18.21 | 3.31 – 40.70 | <0.001 | 3.23 |
|  | φ c p c | 62.69 | 20.86 | 20.78 | 4.96 – 45.53 | <0.001 | 3.01 |
| CRATMO* | φ c p c | 6.43 | 2.64 | 3.66 | 0 – 10.66 | 0.033 | 2.44 |
| FAGUSY | φ t p c | 3.37 | 3.26 | 3.62 | 0 – 14.26 | 0.627 | 1.03 |
|  | φ c p c | 8.95 | 6.52 | 6.89 | 0 – 24.95 | 0.169 | 1.37 |
| FRAXEX* | φ c p c | 5.39 | 3.21 | 3.69 | 0 – 11.87 | 0.119 | 1.68 |
| QUERRO* | φ c p c | 6.05 | 3.26 | 3.68 | 0 – 14.98 | 0.103 | 1.86 |
| SALISP | φ t p c | 13.21 | 11.74 | 11.35 | 0 – 29.16 | 0.495 | 1.13 |
|  | φ c p c | 15.87 | 16.46 | 16.19 | 1.73 – 32.99 | 0.520 | 0.96 |
| SAMBINI | φ t p c | 43.44 | 19.73 | 19.41 | 2.88 – 42.15 | <0.001 | 2.20 |
|  | φ c p c | 54.76 | 24.99 | 24.83 | 8.86 – 50.73 | <0.001 | 2.19 |
| All spp combined | φ t p c | 146.93 | 16.36 | 15.57 | 3.90 – 39.89 | <0.001 | 8.98 |
|  | φ c p c | 150.17 | 21.39 | 20.64 | 5.76 – 50.83 | <0.001 | 7.02 |

* Cannot create the more fully parameterised model, so reduced models have to be used.

So for five species the more fully parameterised model could not be created, of the other five, for three the model broke the assumptions of a CJS model significantly. For the more simplified model, this could be created for all ten species of these four broke the assumptions of a CJS model (ACERPS, CORYAV, CRATMO and SAMBINI).

***Supplement 1b.*** *The output of the bootstrapping exercise on the simplified model to determine model goodness of fit and to estimate the quasi-likelihood parameter used in subsequent analyses of survival.*

|  | φ sapling c, adult c p c | Goodness of fit results estimated from 1000 bootstrap simulations | | | |  |
| --- | --- | --- | --- | --- | --- | --- |
|  | Observed model deviance | Mean model deviance | Median model deviance | Range of model deviance | Probability observed deviance does not differ from simulation | Estimated ^c |
| ACERCA | 7.45 | 2.14 | 0 | 0 – 11.07 | 0.026 | 3.48 |
| ACERPS | 42.14 | 17.08 | 16.19 | 3.13 – 41.89 | <0.001 | 2.47 |
| BETUSP | 12.68 | 8.77 | 8.33 | 0 – 19.54 | 0.090 | 1.44 |
| CORYAV | 88.07 | 31.57 | 31.01 | 11.95 – 64.4 | <0.001 | 2.79 |
| CRATMO | 10.68 | 4.48 | 3.71 | 0 – 14.23 | 0.026 | 2.38 |
| FAGUSY | 12.60 | 10.37 | 10.46 | 0 – 26.18 | 0.255 | 1.22 |
| FRAXEX | 9.49 | 5.33 | 4.60 | 0 – 13.88 | 0.080 | 1.78 |
| QUERRO | 10.16 | 5.12 | 4.44 | 0 – 15.45 | 0.069 | 1.98 |
| SALISP | 27.02 | 23.61 | 22.99 | 7.90 – 48.81 | 0.305 | 1.59 |
| SAMBINI | 74.71 | 35.59 | 35.16 | 11.84 – 62.10 | <0.0001 | 2.12 |

**Supplement 1c**. Model output for each of the 17 models run for each tree species. The models are sorted by quasi-AICc (QAICc). For ACERCA and BETUSP no saplings were recorded and thus models 2, 3, 6, 9, 10, 11, 12, 13, 14, and 17 (as described in table 1) could not be fitted.

ACERCA

|  | Model | QAICc | Delta QAICc | QAICc weight | Model Likelihood | No. Parameters | QDeviance |
| --- | --- | --- | --- | --- | --- | --- | --- |
| 1 | φ c p c | 12.39 | 0.00 | 0.29 | 0.43 | 3 | 6.24 |
| 16 | φ 93-08, 08-12 p c | 16.37 | 5.68 | 0.04 | 0.06 | 5 | 5.98 |
| 7 | φ c sapling growth adult growth p c | 66.56 | 55.87 | 0.00 | 0.00 | 1 | 64.53 |
| 8 | φ c sapling light adult light p c | 68.61 | 57.92 | 0.00 | 0.00 | 2 | 64.53 |
| 5 | φ c sapling c adult DBH p c | 70.69 | 60.00 | 0.00 | 0.00 | 3 | 64.53 |
| 4 | φ c sapling growth adult p c | 70.69 | 60.00 | 0.00 | 0.00 | 3 | 64.53 |
| 15 | φ c sapling DBH growth light adult DBH growth light p c | 81.49 | 70.80 | 0.00 | 0.00 | 8 | 64.53 |

ACERPS

|  | Model | QAICc | Delta QAICc | QAICc weight | Model Likelihood | No. Parameters | QDeviance |
| --- | --- | --- | --- | --- | --- | --- | --- |
| 1 | φ c p c | 59.28 | 0.00 | 0.62 | 1.00 | 3 | 53.12 |
| 2 | φ c sapling DBH adult c p c | 61.06 | 1.79 | 0.25 | 0.41 | 4 | 52.81 |
| 16 | φ 93-08, 08-12 p c | 62.43 | 3.16 | 0.13 | 0.21 | 5 | 52.05 |
| 3 | φ c sapling light adult p c | 309.23 | 249.95 | 0.00 | 0.00 | 3 | 303.08 |
| 10 | φ c sapling DBH adult growth p c | 309.23 | 249.95 | 0.00 | 0.00 | 3 | 303.08 |
| 5 | φ c sapling c adult DBH p c | 309.23 | 249.95 | 0.00 | 0.00 | 3 | 303.08 |
| 4 | φ c sapling growth adult p c | 309.23 | 249.95 | 0.00 | 0.00 | 3 | 303.08 |
| 7 | φ c sapling growth adult growth p c | 311.34 | 252.06 | 0.00 | 0.00 | 4 | 303.08 |
| 9 | φ c sapling DBH adult light p c | 311.34 | 252.06 | 0.00 | 0.00 | 4 | 303.08 |
| 8 | φ c sapling light adult light p c | 311.34 | 252.06 | 0.00 | 0.00 | 4 | 303.08 |
| 13 | φ c sapling growth adult DBH p c | 311.34 | 252.06 | 0.00 | 0.00 | 4 | 303.08 |
| 11 | φ c sapling light adult DBH p c | 311.34 | 252.06 | 0.00 | 0.00 | 4 | 303.08 |
| 14 | φ c sapling growth adult light p c | 311.34 | 252.06 | 0.00 | 0.00 | 4 | 303.08 |
| 12 | φ c sapling light adult growth p c | 311.34 | 252.06 | 0.00 | 0.00 | 4 | 303.08 |
| 6 | φ c sapling DBH adult DBH p c | 313.47 | 254.19 | 0.00 | 0.00 | 5 | 303.08 |
| 17 | φ 93-08, 08-12 sapling DBH adult c p c | 315.63 | 256.35 | 0.00 | 0.00 | 6 | 303.08 |
| 15 | φ c sapling DBH growth light adult DBH growth light p c | 320.03 | 260.75 | 0.00 | 0.00 | 8 | 303.08 |

BETUSP

|  | Model | QAICc | Delta QAICc | QAICc weight | Model Likelihood | No. Parameters | QDeviance |
| --- | --- | --- | --- | --- | --- | --- | --- |
| 1 | φ c p c | 60.23 | 0.00 | 0.21 | 0.28 | 3 | 54.07 |
| 16 | φ 93-08, 08-12 p c | 62.71 | 5.03 | 0.06 | 0.08 | 5 | 52.32 |
| 5 | φ c sapling c adult DBH p c | 141.50 | 83.82 | 0.00 | 0.00 | 3 | 135.34 |
| 4 | φ c sapling growth adult p c | 144.65 | 86.97 | 0.00 | 0.00 | 3 | 138.50 |
| 7 | φ c sapling growth adult growth p c | 146.75 | 89.07 | 0.00 | 0.00 | 4 | 138.49 |
| 8 | φ c sapling light adult light p c | 146.75 | 89.07 | 0.00 | 0.00 | 4 | 138.50 |
| 15 | φ c sapling DBH growth light adult DBH growth light p c | 149.10 | 91.42 | 0.00 | 0.00 | 8 | 132.15 |

CORYAV

|  | Model | QAICc | Delta QAICc | QAICc weight | Model Likelihood | No. Parameters | QDeviance |
| --- | --- | --- | --- | --- | --- | --- | --- |
| 2 | φ c sapling DBH adult c p c | 58.49 | 0.00 | 0.66 | 1.00 | 3 | 52.34 |
| 1 | φ c p c | 60.04 | 1.54 | 0.30 | 0.46 | 3 | 53.88 |
| 16 | φ 93-08, 08-12 p c | 64.27 | 5.78 | 0.04 | 0.06 | 5 | 53.88 |
| 10 | φ c sapling DBH adult growth p c | 122.42 | 63.93 | 0.00 | 0.00 | 3 | 116.27 |
| 5 | φ c sapling c adult DBH p c | 122.42 | 63.93 | 0.00 | 0.00 | 3 | 116.27 |
| 11 | φ c sapling light adult DBH p c | 122.42 | 63.93 | 0.00 | 0.00 | 3 | 116.27 |
| 4 | φ c sapling growth adult p c | 122.42 | 63.93 | 0.00 | 0.00 | 3 | 116.27 |
| 3 | φ c sapling light adult p c | 122.42 | 63.93 | 0.00 | 0.00 | 3 | 116.27 |
| 6 | φ c sapling DBH adult DBH p c | 124.53 | 66.04 | 0.00 | 0.00 | 4 | 116.27 |
| 13 | φ c sapling growth adult DBH p c | 124.53 | 66.04 | 0.00 | 0.00 | 4 | 116.27 |
| 9 | φ c sapling DBH adult light p c | 124.53 | 66.04 | 0.00 | 0.00 | 4 | 116.27 |
| 7 | φ c sapling growth adult growth p c | 124.53 | 66.04 | 0.00 | 0.00 | 4 | 116.27 |
| 14 | φ c sapling growth adult light p c | 124.53 | 66.04 | 0.00 | 0.00 | 4 | 116.27 |
| 12 | φ c sapling light adult growth p c | 124.53 | 66.04 | 0.00 | 0.00 | 4 | 116.27 |
| 8 | φ c sapling light adult light p c | 124.53 | 66.04 | 0.00 | 0.00 | 4 | 116.27 |
| 17 | φ 93-08, 08-12 sapling DBH adult c p c | 128.82 | 70.33 | 0.00 | 0.00 | 6 | 116.27 |
| 15 | φ c sapling DBH growth light adult DBH growth light p c | 133.22 | 74.73 | 0.00 | 0.00 | 8 | 116.27 |

CRATMO

|  | Model | QAICc | Delta QAICc | QAICc weight | Model Likelihood | No. Parameters | QDeviance |
| --- | --- | --- | --- | --- | --- | --- | --- |
| 2 | φ c sapling DBH adult c p c | 24.90 | 0.00 | 0.46 | 1.00 | 3 | 18.74 |
| 1 | φ c p c | 25.06 | 0.16 | 0.42 | 0.92 | 3 | 18.91 |
| 16 | φ 93-08, 08-12 p c | 27.56 | 2.66 | 0.12 | 0.26 | 5 | 17.17 |
| 10 | φ c sapling DBH adult growth p c | 115.91 | 91.02 | 0.00 | 0.00 | 2 | 111.84 |
| 5 | φ c sapling c adult DBH p c | 115.91 | 91.02 | 0.00 | 0.00 | 2 | 111.84 |
| 4 | φ c sapling growth adult p c | 117.99 | 93.09 | 0.00 | 0.00 | 3 | 111.84 |
| 3 | φ c sapling light adult p c | 117.99 | 93.09 | 0.00 | 0.00 | 3 | 111.84 |
| 12 | φ c sapling light adult growth p c | 117.99 | 93.09 | 0.00 | 0.00 | 3 | 111.84 |
| 11 | φ c sapling light adult DBH p c | 120.09 | 95.20 | 0.00 | 0.00 | 4 | 111.84 |
| 6 | φ c sapling DBH adult DBH p c | 120.09 | 95.20 | 0.00 | 0.00 | 4 | 111.84 |
| 13 | φ c sapling growth adult DBH p c | 120.09 | 95.20 | 0.00 | 0.00 | 4 | 111.84 |
| 9 | φ c sapling DBH adult light p c | 120.09 | 95.20 | 0.00 | 0.00 | 4 | 111.84 |
| 7 | φ c sapling growth adult growth p c | 120.09 | 95.20 | 0.00 | 0.00 | 4 | 111.84 |
| 8 | φ c sapling light adult light p c | 120.09 | 95.20 | 0.00 | 0.00 | 4 | 111.84 |
| 14 | φ c sapling growth adult light p c | 122.23 | 97.33 | 0.00 | 0.00 | 5 | 111.84 |
| 17 | φ 93-08, 08-12 sapling DBH adult c p c | 124.39 | 99.49 | 0.00 | 0.00 | 6 | 111.84 |
| 15 | φ c sapling DBH growth light adult DBH growth light p c | 128.79 | 103.89 | 0.00 | 0.00 | 8 | 111.84 |

FAGUSY

|  | Model | QAICc | Delta QAICc | QAICc weight | Model Likelihood | No. Parameters | QDeviance |
| --- | --- | --- | --- | --- | --- | --- | --- |
| 2 | φ c sapling DBH adult c p c | 33.57 | 0.00 | 1.00 | 1.00 | 3 | 27.42 |
| 1 | φ c p c | 52.96 | 19.39 | 0.00 | 0.00 | 3 | 46.81 |
| 16 | φ 93-08, 08-12 p c | 57.20 | 23.63 | 0.00 | 0.00 | 5 | 46.81 |
| 10 | φ c sapling DBH adult growth p c | 255.29 | 221.72 | 0.00 | 0.00 | 2 | 251.22 |
| 6 | φ c sapling DBH adult DBH p c | 255.29 | 221.72 | 0.00 | 0.00 | 2 | 251.22 |
| 9 | φ c sapling DBH adult light p c | 255.29 | 221.72 | 0.00 | 0.00 | 2 | 251.22 |
| 15 | φ c sapling DBH growth light adult DBH growth light p c | 255.29 | 221.72 | 0.00 | 0.00 | 2 | 251.22 |
| 5 | φ c sapling c adult DBH p c | 267.13 | 233.55 | 0.00 | 0.00 | 1 | 265.10 |
| 17 | φ 93-08, 08-12 sapling DBH adult c p c | 268.72 | 235.15 | 0.00 | 0.00 | 2 | 264.65 |
| 3 | φ c sapling light adult p c | 269.18 | 235.61 | 0.00 | 0.00 | 2 | 265.10 |
| 11 | φ c sapling light adult DBH p c | 269.18 | 235.61 | 0.00 | 0.00 | 2 | 265.10 |
| 8 | φ c sapling light adult light p c | 269.18 | 235.61 | 0.00 | 0.00 | 2 | 265.10 |
| 4 | φ c sapling growth adult p c | 271.25 | 237.68 | 0.00 | 0.00 | 3 | 265.09 |
| 13 | φ c sapling growth adult DBH p c | 271.25 | 237.68 | 0.00 | 0.00 | 3 | 265.09 |
| 7 | φ c sapling growth adult growth p c | 271.25 | 237.68 | 0.00 | 0.00 | 3 | 265.09 |
| 14 | φ c sapling growth adult light p c | 271.25 | 237.68 | 0.00 | 0.00 | 3 | 265.09 |
| 12 | φ c sapling light adult growth p c | 273.36 | 239.79 | 0.00 | 0.00 | 4 | 265.10 |

FRAXEX

|  | Model | QAICc | Delta QAICc | QAICc weight | Model Likelihood | No. Parameters | QDeviance |
| --- | --- | --- | --- | --- | --- | --- | --- |
| 2 | φ c sapling DBH adult c p c | 42.03 | 0.00 | 0.52 | 1.00 | 3 | 35.88 |
| 1 | φ c p c | 42.60 | 0.57 | 0.39 | 0.75 | 3 | 36.45 |
| 16 | φ 93-08, 08-12 p c | 45.38 | 3.35 | 0.10 | 0.19 | 5 | 35.00 |
| 17 | φ 93-08, 08-12 sapling DBH adult c p c | 437.88 | 395.85 | 0.00 | 0.00 | 2 | 433.80 |
| 10 | φ c sapling DBH adult growth p c | 440.73 | 398.70 | 0.00 | 0.00 | 3 | 434.58 |
| 5 | φ c sapling c adult DBH p c | 440.73 | 398.70 | 0.00 | 0.00 | 3 | 434.58 |
| 3 | φ c sapling light adult p c | 440.73 | 398.70 | 0.00 | 0.00 | 3 | 434.58 |
| 4 | φ c sapling growth adult p c | 440.73 | 398.70 | 0.00 | 0.00 | 3 | 434.58 |
| 6 | φ c sapling DBH adult DBH p c | 442.84 | 400.81 | 0.00 | 0.00 | 4 | 434.58 |
| 9 | φ c sapling DBH adult light p c | 442.84 | 400.81 | 0.00 | 0.00 | 4 | 434.58 |
| 11 | φ c sapling light adult DBH p c | 442.84 | 400.81 | 0.00 | 0.00 | 4 | 434.58 |
| 8 | φ c sapling light adult light p c | 442.84 | 400.81 | 0.00 | 0.00 | 4 | 434.58 |
| 13 | φ c sapling growth adult DBH p c | 442.84 | 400.81 | 0.00 | 0.00 | 4 | 434.58 |
| 7 | φ c sapling growth adult growth p c | 442.84 | 400.81 | 0.00 | 0.00 | 4 | 434.58 |
| 14 | φ c sapling growth adult light p c | 442.84 | 400.81 | 0.00 | 0.00 | 4 | 434.58 |
| 12 | φ c sapling light adult growth p c | 442.84 | 400.81 | 0.00 | 0.00 | 4 | 434.58 |
| 15 | φ c sapling DBH growth light adult DBH growth light p c | 451.53 | 409.50 | 0.00 | 0.00 | 8 | 434.58 |

QUERRO

|  | Model | QAICc | Delta QAICc | QAICc weight | Model Likelihood | No. Parameters | QDeviance |
| --- | --- | --- | --- | --- | --- | --- | --- |
| 2 | φ c sapling DBH adult c p c | 33.09 | 0.00 | 0.52 | 1.00 | 3 | 26.94 |
| 1 | φ c p c | 33.56 | 0.47 | 0.41 | 0.79 | 3 | 27.40 |
| 16 | φ 93-08, 08-12 p c | 37.30 | 4.21 | 0.06 | 0.12 | 5 | 26.91 |
| 17 | φ 93-08, 08-12 sapling DBH adult c p c | 167.91 | 134.82 | 0.00 | 0.00 | 2 | 163.84 |
| 5 | φ c sapling c adult DBH p c | 167.91 | 134.82 | 0.00 | 0.00 | 2 | 163.84 |
| 9 | φ c sapling DBH adult light p c | 167.91 | 134.82 | 0.00 | 0.00 | 2 | 163.84 |
| 8 | φ c sapling light adult light p c | 167.91 | 134.82 | 0.00 | 0.00 | 2 | 163.84 |
| 3 | φ c sapling light adult p c | 169.99 | 136.90 | 0.00 | 0.00 | 3 | 163.84 |
| 4 | φ c sapling growth adult p c | 169.99 | 136.90 | 0.00 | 0.00 | 3 | 163.84 |
| 6 | φ c sapling DBH adult DBH p c | 172.09 | 139.00 | 0.00 | 0.00 | 4 | 163.84 |
| 11 | φ c sapling light adult DBH p c | 172.09 | 139.00 | 0.00 | 0.00 | 4 | 163.84 |
| 13 | φ c sapling growth adult DBH p c | 172.09 | 139.00 | 0.00 | 0.00 | 4 | 163.84 |
| 7 | φ c sapling growth adult growth p c | 172.09 | 139.00 | 0.00 | 0.00 | 4 | 163.84 |
| 14 | φ c sapling growth adult light p c | 172.09 | 139.00 | 0.00 | 0.00 | 4 | 163.84 |
| 12 | φ c sapling light adult growth p c | 172.09 | 139.00 | 0.00 | 0.00 | 4 | 163.84 |
| 10 | φ c sapling DBH adult growth p c | 174.22 | 141.14 | 0.00 | 0.00 | 5 | 163.84 |
| 15 | φ c sapling DBH growth light adult DBH growth light p c | 180.79 | 147.70 | 0.00 | 0.00 | 8 | 163.84 |

SALISP

|  | Model | QAICc | Delta QAICc | QAICc weight | Model Likelihood | No. Parameters | QDeviance |
| --- | --- | --- | --- | --- | --- | --- | --- |
| 16 | φ 93-08, 08-12 p c | 49.73 | 0.00 | 0.71 | 1.00 | 4 | 41.47 |
| 1 | φ c p c | 52.11 | 2.38 | 0.22 | 0.30 | 3 | 45.96 |
| 2 | φ c sapling DBH adult c p c | 54.19 | 4.47 | 0.08 | 0.11 | 4 | 45.94 |
| 9 | φ c sapling DBH adult light p c | 67.79 | 18.06 | 0.00 | 0.00 | 3 | 61.64 |
| 8 | φ c sapling light adult light p c | 67.85 | 18.12 | 0.00 | 0.00 | 3 | 61.69 |
| 7 | φ c sapling growth adult growth p c | 67.85 | 18.12 | 0.00 | 0.00 | 3 | 61.69 |
| 14 | φ c sapling growth adult light p c | 67.85 | 18.12 | 0.00 | 0.00 | 3 | 61.69 |
| 12 | φ c sapling light adult growth p c | 67.85 | 18.12 | 0.00 | 0.00 | 3 | 61.69 |
| 5 | φ c sapling c adult DBH p c | 68.39 | 18.66 | 0.00 | 0.00 | 3 | 62.23 |
| 10 | φ c sapling DBH adult growth p c | 68.39 | 18.67 | 0.00 | 0.00 | 3 | 62.24 |
| 3 | φ c sapling light adult p c | 68.45 | 18.72 | 0.00 | 0.00 | 3 | 62.30 |
| 4 | φ c sapling growth adult p c | 68.45 | 18.72 | 0.00 | 0.00 | 3 | 62.30 |
| 17 | φ 93-08, 08-12 sapling DBH adult c p c | 69.97 | 20.25 | 0.00 | 0.00 | 5 | 59.58 |
| 6 | φ c sapling DBH adult DBH p c | 70.39 | 20.66 | 0.00 | 0.00 | 4 | 62.13 |
| 11 | φ c sapling light adult DBH p c | 70.45 | 20.72 | 0.00 | 0.00 | 4 | 62.19 |
| 13 | φ c sapling growth adult DBH p c | 70.45 | 20.72 | 0.00 | 0.00 | 4 | 62.19 |
| 15 | φ c sapling DBH growth light adult DBH growth light p c | 71.91 | 22.18 | 0.00 | 0.00 | 5 | 61.52 |

SAMBINI

|  | Model | QAICc | Delta QAICc | QAICc weight | Model Likelihood | No. Parameters | QDeviance |
| --- | --- | --- | --- | --- | --- | --- | --- |
| 1 | φ c p c | 89.02 | 0.00 | 0.68 | 1.00 | 3 | 82.87 |
| 2 | φ c sapling DBH adult c p c | 91.09 | 2.07 | 0.24 | 0.36 | 4 | 82.84 |
| 16 | φ 93-08, 08-12 p c | 93.22 | 4.19 | 0.08 | 0.12 | 5 | 82.83 |
| 17 | φ 93-08, 08-12 sapling DBH adult c p c | 133.89 | 44.87 | 0.00 | 0.00 | 3 | 127.73 |
| 10 | φ c sapling DBH adult growth p c | 134.97 | 45.95 | 0.00 | 0.00 | 3 | 128.82 |
| 5 | φ c sapling c adult DBH p c | 135.48 | 46.45 | 0.00 | 0.00 | 3 | 129.32 |
| 4 | φ c sapling growth adult p c | 135.78 | 46.76 | 0.00 | 0.00 | 3 | 129.63 |
| 3 | φ c sapling light adult p c | 135.78 | 46.76 | 0.00 | 0.00 | 3 | 129.63 |
| 6 | φ c sapling DBH adult DBH p c | 136.77 | 47.75 | 0.00 | 0.00 | 4 | 128.51 |
| 9 | φ c sapling DBH adult light p c | 137.04 | 48.01 | 0.00 | 0.00 | 4 | 128.78 |
| 13 | φ c sapling growth adult DBH p c | 137.58 | 48.56 | 0.00 | 0.00 | 4 | 129.32 |
| 11 | φ c sapling light adult DBH p c | 137.58 | 48.56 | 0.00 | 0.00 | 4 | 129.32 |
| 14 | φ c sapling growth adult light p c | 137.85 | 48.82 | 0.00 | 0.00 | 4 | 129.59 |
| 8 | φ c sapling light adult light p c | 137.85 | 48.82 | 0.00 | 0.00 | 4 | 129.59 |
| 7 | φ c sapling growth adult growth p c | 137.88 | 48.86 | 0.00 | 0.00 | 4 | 129.63 |
| 12 | φ c sapling light adult growth p c | 137.88 | 48.86 | 0.00 | 0.00 | 4 | 129.63 |
| 15 | φ c sapling DBH growth light adult DBH growth light p c | 144.98 | 55.96 | 0.00 | 0.00 | 8 | 128.03 |
