# Supplementary material for: Effects of growth rate, size, and light availability on tree survival across life stages: a demographic analysis accounting for missing values and small sample sizes
Source: BMC Ecol. 2015 Feb 28;15:6. doi: 10.1186/s12898-015-0038-8 (PMC4465470; doi:10.1186/s12898-015-0038-8)

**Supplement 2 a.** Effects of growth rate, light, and DBH on tree survival. These results derive from the second best model applied to the data assuming all trees were equivalent (model 4 in table 7 in the main document). As plotting the effects of al three covariates on survival would require four dimensions, we have plotted model outputs on the effect of each covariate (growth rate, light, and DBH) for combinations of high and low values of the remaining two covariates resulting in 3 x 4 = 12 graphs in total. The first four graphs show the effects of growth rate on survival for low light in small trees, low light in large trees, high light in small trees, and high light in large trees. The next four graphs show the effects of light on survival for low growth in small trees, low growth in large trees, high growth in small trees, and high growth in large trees. The last four graphs show the effects of size (DBH) on survival for low light low growth rates, low light high growth rates, high light low growth rates, and high light high growth rates. Growth rates and light availability are measured in % and thus have no units, tree size (DBH) is measured in cm – see methods for details.

**Survival as a factor growth rates for small (10 cm DBH) and for large sized tress (230 cm DBH), in low light (0.1% or 0.001) and high light (0.055 or 5.5%) conditions.**

**Survival as a function of light availability for small (10 cm DBH) and large tress (230 cm DBH) for low growth rates (10% or 0.1) and high growth raters (80% or 0.8).**

**Survival as a function of tree size (DBH) for low growth (0.1 or 10%) and high growth (0.8 or 80%), in low light (0.001 or 0.1%) and high light (0.055 or 5.5%) availability.**

Overall, high mortality rates are associated with both high and low growth rates (first four figures), while mortality rates are dropping (survival rates are increasing) for intermediate growth vales. This result is more pronounced on high light rather than in low light availability and within this effect small sized trees have lower survival rates than larger sized ones. Note that Elder a subcanopy species, as well as Ash, and Beech that are canopy species, were relatively insensitive to light and size, having a constant survival rate.

In terms of survival as a function of light availability (next four figures), Willow and Birch tress exhibited lower survival rates with increasing light availability for small sized low growing trees. Small sized individuals of Hazel and Hawthorn had low survival rates when growing fast, but overall all species except Willow, had decreasing survival rates with increasing light availability when growing fast.

In terms of survival as a function of size, slow growing tress appear to be insensitive to light (figures growing at 0.1 in light of 0.001 and 0.055 as well as figures growing at 0.8 in light of 0.001 and 0.055). Most species at low growth rates (0.1) had constant survival in terms of DBH except Willow that larger individuals had higher survival rates and Elder and Willow that had lower survival rates with increased size.

**Supplement 2b.** Survival probabilities as in figure 3 of the main text but expanded in 0.94 – 1.0 survival probability.


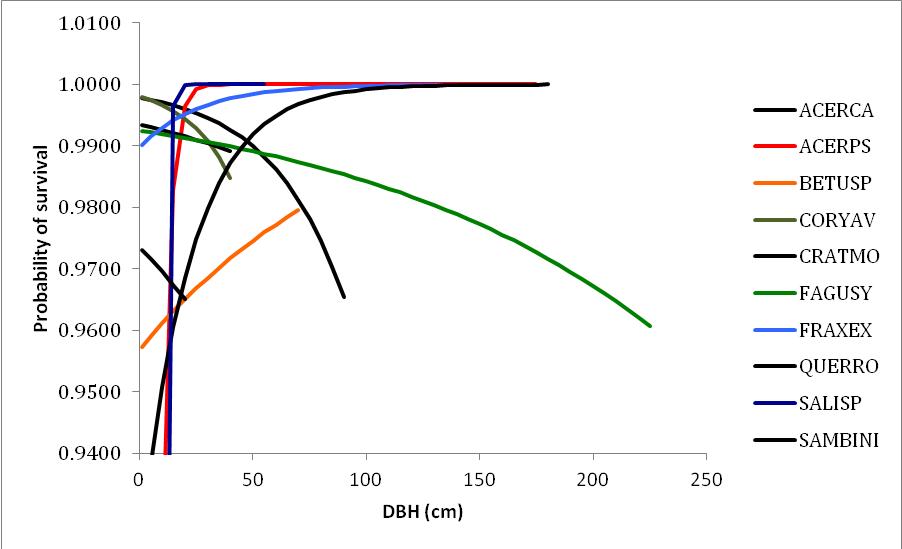

Supplement: Additional file 2: — a. Effects of growth rate, light, and DBH on tree survival. These results derive from the second best model applied to the data assuming all trees were equivalent (model 4 in table seven in the main document). As plotting the effects of al three covariates on survival would require four dimensions, we have plotted model outputs on the effect of each covariate (growth rate, light, and DBH) for combinations of high and low values of the remaining two covariates resulting in 3 × 4 = 12 graphs in total. The first four graphs show the effects of growth rate on survival for low light in small trees, low light in large trees, high light in small trees, and high light in large trees. The next four graphs show the effects of light on survival for low growth in small trees, low growth in large trees, high growth in small trees, and high growth in large trees. The last four graphs show the effects of size (DBH) on survival for low light low growth rates, low light high growth rates, high light low growth rates, and high light high growth rates. Growth rates and light availability are measured in % and thus have no units, tree size (DBH) is measured in cm – see methods for details. b. Survival probabilities as in figure 3 of the main text but expanded in 0.94 – 1.0 survival probability. [file 12898_2015_38_MOESM2_ESM.docx]
